# TNFRSF13B is a potential contributor to prostate cancer

**DOI:** 10.1186/s12935-022-02590-2

**Published:** 2022-05-06

**Authors:** Chia-Yang Li, Shu-Pin Huang, Yei-Tsung Chen, Hsin-En Wu, Wei-Chung Cheng, Chao-Yuan Huang, Chia-Cheng Yu, Victor C. Lin, Jiun-Hung Geng, Te-Ling Lu, Bo-Ying Bao

**Affiliations:** 1 Graduate Institute of Medicine, College of Medicine, Kaohsiung Medical University, Kaohsiung, 807 Taiwan; 2 Department of Medical Research, Kaohsiung Medical University Hospital, Kaohsiung, 807 Taiwan; 3 Department of Urology, Kaohsiung Medical University Hospital, Kaohsiung, 807 Taiwan; 4 Graduate Institute of Clinical Medicine, College of Medicine, Kaohsiung Medical University, Kaohsiung, 807 Taiwan; 5 Department of Urology, Faculty of Medicine, College of Medicine, Kaohsiung Medical University, Kaohsiung, 807 Taiwan; 6 Program in Environmental and Occupational Medicine, College of Medicine, Kaohsiung Medical University, Kaohsiung, 807 Taiwan; 7 Department of Life Sciences, Institute of Genome Sciences, National Yang Ming Chiao Tung University, Taipei, 112 Taiwan; 8 Graduate Institute of Biomedical Science, China Medical University, Taichung, 40403 Taiwan; 9 Department of Urology, College of Medicine, National Taiwan University Hospital, National Taiwan University, Taipei, 100 Taiwan; 10 Division of Urology, Department of Surgery, Kaohsiung Veterans General Hospital, Kaohsiung, 813 Taiwan; 11 Department of Urology, School of Medicine, National Yang-Ming University, Taipei, 112 Taiwan; 12 Department of Pharmacy, Tajen University, Pingtung, 907 Taiwan; 13 Department of Urology, E-Da Hospital, Kaohsiung, 824 Taiwan; 14 School of Medicine for International Students, I-Shou University, Kaohsiung, 840 Taiwan; 15 Department of Urology, Kaohsiung Municipal Hsiao-Kang Hospital, 812 Kaohsiung, Taiwan; 16 Department of Pharmacy, China Medical University, 100 Jingmao Road Section 1, Taichung, 406 Taiwan; 17 Sex Hormone Research Center, China Medical University Hospital, Taichung, 404 Taiwan; 18 Department of Nursing, Asia University, Taichung, 413 Taiwan

**Keywords:** Immunodeficiency, Prostate cancer, TNFRSF13B, Biochemical recurrence, Prognosis, Biomarker

## Abstract

**Background:**

Immunodeficiencies are genetic diseases known to predispose an individual to cancer owing to defective immunity towards malignant cells. However, the link between immunodeficiency and prostate cancer progression remains unclear. Therefore, the aim of this study was to evaluate the effects of common genetic variants among eight immunodeficiency pathway-related genes on disease recurrence in prostate cancer patients treated with radical prostatectomy.

**Methods:**

Genetic and bioinformatic analyses on 19 haplotype-tagging single-nucleotide polymorphisms in eight immunodeficiency pathway-related genes were conducted in 458 patients with prostate cancer after receiving radical prostatectomy. Furthermore, the *TNFRSF13B* was knocked down in 22Rv1 and PC-3 human prostate cancer cell lines via transfecting short hairpin RNAs and cell proliferation and colony formation assays were performed. The molecular mechanisms underlying the effects of *TNFRSF13B* were further explored by microarray gene expression profiling.

**Results:**

*TNFRSF13B* rs4792800 was found to be significantly associated with biochemical recurrence even after adjustment for clinical predictors and false discovery rate correction (adjusted hazard ratio 1.78, 95% confidence interval 1.16–2.71, *p* = 0.008), and the G allele was associated with higher *TNFRSF13B* expression (*p* = 0.038). Increased *TNFRSF13B* expression suggested poor prognosis in four independent prostate cancer datasets. Furthermore, silencing *TNFRSF13B* expression resulted in decreased colony formation of 22Rv1 and PC-3 cells through modulating the cell cycle and p53 signalling pathways.

**Conclusions:**

The present study suggests the potential role of immunodeficiency pathway-related genes, primarily *TNFRSF13B*, in prostate cancer progression.

**Supplementary information:**

The online version contains supplementary material available at 10.1186/s12935-022-02590-2.

## Introduction

Prostate cancer is a common malignant tumour which occurs in men worldwide, with estimated 1,414,259 new cases and 375,304 deaths in 2020 [1]. With the advancements in prostate-specific antigen (PSA) screening, most prostate cancers are detected at the regional stages (clinically localised disease). Radical prostatectomy is a common treatment for localised prostate cancer with intermediate or high risk of disease progression. However, 20–40% of the patients who undergo radical prostatectomy are reported to experience biochemical recurrence (BCR) within 10 years [2, 3]. Therefore, understanding the pathological mechanisms and identification of potent prognostic biomarkers for BCR are needed to identify patients at high-risk of disease development and guide treatment decisions.

The immune system plays a key role in surveillance against cancer, but some tumour cells evolve to escape immune elimination [4]. Cancer cells use various immune escape mechanisms including the loss of antigenicity through defective antigen presentation in a peptide–major histocompatibility complex [5], loss of immunogenicity through the upregulation of the immunoinhibitory molecule, programmed death-ligand 1, or secretion of immunosuppressive cytokines [6], and orchestrating an immunosuppressive microenvironment through recruiting immunosuppressive leukocytes [7]. Abundant evidence indicates that immunodeficiencies are genetic or acquired disorders that predisposes an individual to cancer. The incidence of malignancies in children with congenital immunodeficiency has been estimated to be 4%, which is nearly 10,000 times higher than that in healthy controls of similar age. Moreover, children with acquired immunodeficiencies are at a higher risk of developing a malignancy than their healthy counterparts [8]. Organ transplant recipients under immunosuppressive treatments are at a 20-fold increased risk of developing de novo carcinoma [9]. Furthermore, patients with immunodeficiency were reported to have a 13-fold increase in the incidence of lymphomas and a 6-fold increase in the incidence of gastric cancers [10, 11]. Recent reports have also described a relatively early onset of breast and prostate cancers in patients with immunodeficiency [12, 13].

Previous genetic association studies mainly focused on evaluating the risk of developing prostate cancer rather than clinical outcomes after treatments [14–16]. However, no study has yet systematically investigated the effect of gene variants related to immunodeficiency on prostate cancer progression. In the present study, we first conducted a genetic analysis to evaluate the effects of common variants among eight immunodeficiency pathway-related genes on disease recurrence in 458 patients with prostate cancer after receiving radical prostatectomy. Further, gene knockdown experiments were performed in human prostate cancer cell lines, and the consequent transcriptome changes were evaluated to assess the contributions of specific genes and associated pathways to prostate cancer.

## Methods

### Patient recruitment and data collection

Four hundred and fifty-eight patients with histologically confirmed prostate cancer receiving radical prostatectomy were recruited from three medical centres across Taiwan, namely, the Kaohsiung Medical University Hospital, Kaohsiung Veterans General Hospital, and National Taiwan University Hospital, as described previously [17]. Clinical and prognostic data for patients were collected through medical chart review, and the study was approved by the institutional review board of Kaohsiung Medical University Hospital (KMUHIRB-2013132). Written informed consent was obtained from each participant, and the study was conducted in accordance with the Declaration of Helsinki. BCR-free survival was defined as the interval between time of radical prostatectomy and detection of two consecutive PSA levels ≥ 0.2 ng/mL [18–21]. Peripheral blood was collected from all study participants, and genomic DNA was extracted using the QIAamp DNA Blood Mini Kit (Qiagen, Valencia, CA, USA), and stored at ‒ 20 ºC until further use.

### Single-nucleotide polymorphism (SNP) selection and genotyping

A gene list was compiled using the Kyoto Encyclopaedia of Genes and Genomes (KEGG) database and a literature review was performed to refine potentially cancer-related genes in the primary immunodeficiency pathway. Eight genes, namely, CD3d, CD3e, CD8a, and CD19 molecules, class II major histocompatibility complex transactivator, inducible T cell costimulator, tumour necrosis factor receptor superfamily member 13B (*TNFRSF13B*), and zeta chain of T cell receptor associated protein kinase 70, were identified. Haplotype-tagging SNPs (htSNPs) in these genes were selected using SNPinfo [22] with minor allele frequency > 0.05 in the HapMap Chinese Han Beijing population and a pairwise linkage disequilibrium *r*^2^ > 0.8. Genotyping was performed at the National Centre for Genome Medicine, Taiwan, using the Agena Bioscience (San Diego, CA, USA) iPLEX matrix-assisted laser desorption/ionization time-of-flight mass-spectrometry technology, as described previously [23]. Ten blinded quality control samples were included in the genotyping assays, and the concordance rate was 97.4%. The average call rate was 99.5%, and three SNPs that deviated from Hardy–Weinberg equilibrium (*p* < 0.05) were removed, leaving a total of 19 htSNPs for further analyses.

### Bioinformatics analysis

HaploReg v4.1 was used to identify proxy variants in strong linkage disequilibrium with the risk SNP, rs4792800, and to annotate their potential regulatory functions [24]. The Genotype-Tissue Expression (GTEx) portal was used to assess the expression quantitative trait locus effect between rs4792800 and *TNFRSF13B* [25]. The effects of *TNFRSF13B* expression on the prognosis of prostate cancer was compared using publicly available gene expression datasets from Jain et al. [26], Taylor et al. [27], Long et al. [28], and The Cancer Genome Atlas (TCGA) [29].

### Cell culture and transfection

Human prostate cancer cell lines, 22Rv1 and PC-3, were purchased from the Bioresource Collection and Research Center (Hsinchu, Taiwan) and cultured in RPMI and F12 media (Corning, Corning, NY, USA), respectively. All media were supplemented with 10% heat-inactivated foetal bovine serum, 100 U/mL penicillin, and 0.1 mg/mL streptomycin (Corning, Corning, NY, USA). Cells were maintained in a humidified incubator with 5% CO_2_ at 37 °C. For *TNFRSF13B* knockdown in both 22Rv1 and PC-3 cells, the lentiviral pLKO.1 empty vector and human *TNFRSF13B* targeting short hairpin RNAs (shRNAs) were obtained from the National RNAi Core Facility (Academia Sinica, Taipei, Taiwan) and the target sequence was as follows: 5ʹ-ACAATTCAGACAACTCGGGAA-3ʹ. To generate the lentivirus containing specific shRNA, HEK293T cells were co-transfected with the packaging plasmid (pCMV-Δ8.9 plasmid with *Gag* and *Pol* genes), the envelope plasmid (pMD.G plasmid with *VSV-G*), and pLKO.1-shTNFRSF13B or pLKO.1 empty vector for 24 h using Lipofectamine 2000 (Invitrogen, Carlsbad, CA, USA). The culture medium containing lentivirus was harvested and the 22Rv1 and PC-3 cells were transfected with the lentivirus for 24 h. The cells were then selected in a medium containing puromycin (2 µg/mL).

### Western blot analysis

Cells were washed with phosphate-buffered saline (PBS) and incubated in RIPA lysis buffer supplemented with protease inhibitors (Sigma-Aldrich, St. Louis, MO, USA). Protein concentrations were measured using bicinchoninic acid protein assay (Thermo Fisher Scientific, Waltham, MA, USA). Equal amounts of proteins were separated using sodium dodecyl sulphate–polyacrylamide gel electrophoresis and then transferred onto polyvinylidene difluoride membranes (Millipore, Billerica, MA, USA). The blots were blocked with 5% fat-free milk in PBS containing 0.5% Tween-20 for 1 h at room temperature and incubated with antibodies against TNFRSF13B (1:1000, PA1-41199, Thermo Fisher Scientific, Waltham, MA, USA), p53 (1:1000, sc-126, Santa Cruz Biotechnology, Dallas, TX, USA), and β-tubulin (1:1000, MA5-16308, Thermo Fisher Scientific, Waltham, MA, USA) overnight at 4 °C. After washing with PBS three times, the blots were incubated with peroxidase-conjugated secondary antibody (1:5000) for 1 h at room temperature. The blots were visualised with enhanced chemiluminescence substrate (Thermo Fisher Scientific, Waltham, MA, USA) and detected using the Bio-Rad ChemiDoc XRS + system (Bio-Rad Laboratories, Hercules, CA, USA).

## Cell proliferation assay

Cells were seeded at a concentration of 5 × 10^3^ cells/mL into a 96-well plate and cultured for 4 d. Thereafter, 20 µL of 5 mg/mL 3-(4,5-dimethylthiazol-2-yl)-2,5 diphenyltetrazolium bromide (MTT) solution (Sigma-Aldrich, St. Louis, MO, USA) was added to each well and incubated for 4 h at 37 °C. Afterwards, the supernatants were removed and 100 µL dimethylsulfoxide was added to each well to dissolve the precipitate. The absorbance was measured at 570 nm using a microplate reader (BioTek Instruments, Winooski, VT, USA). Cell proliferation activity was calculated based on the absorbance ratios.

### Colony formation assay

Cells were seeded at a concentration of 1 × 10^3^ cells/mL in 10-cm plates (Corning, Corning, NY, USA) and cultured for three weeks. Cells were fixed with 4% paraformaldehyde for 10 min. Wells were rinsed with PBS and colonies were stained with crystal violet solution (0.05% crystal violet, 1% formaldehyde, 1% methanol and 1× PBS) for 10 min. Excess stain was removed by washing repeatedly with PBS. The number of colonies was quantified using the ImageJ software according to a previous study [30].

### Microarray analysis

Total RNA was isolated using TRIzol reagent according to the manufacturer’s protocol (Thermo Fisher Scientific, Waltham, MA, USA). The quality of isolated total RNA was assessed by the Agilent 2100 bioanalyzer (Agilent, Palo Alto, CA, USA). The total RNA was then amplified, labelled, and hybridised using the Clariom D platform (Affymetrix, Santa Clara, CA, USA) following the manufacturer’s instructions. Briefly, cDNA preparation and biotin labelling were performed using the Affymetrix GeneChip WT Pico kit and then purified using an Affymetrix magnetic bead protocol. The Affymetrix GeneChip hybridisation, wash, and stain kit was then used for array processing. Arrays were incubated for 16 h in an Affymetrix GeneChip 645 hybridization oven at 45 °C with rotation at 60 rpm. The chips were subsequently scanned with Affymetrix GeneChip Scanner 3000. Raw data were analysed using Affymetrix Expression Console and Transcriptome Analysis Console software prior to downstream analysis. The criterion of significantly differentially expressed (SDE) genes was set at a fold change ≥ 2 or ≤ 2 between the *TNFRSF13B* knockdown and control groups. Functional annotation of the SDE genes commonly expressed in both PC-3 and 22Rv1 cells was performed following the methodology of our previous study [31]. Both the KEGG pathway database [32] and the Reactome pathway knowledgebase [33] were used to annotate genes with their associated functions. The microarray data are available in the Gene Expression Omnibus (GSE196746).

### Statistical analysis

The association between the clinicopathological characteristics of the patients, SNPs in different genetic models (including additive, dominant, and recessive), and BCR-free survival were assessed using Cox proportional hazard regression. False discovery rate (FDR)-adjusted *q* values were calculated to account for multiple comparisons [34]. Kaplan–Meier analyses and log-rank tests were used to analyse the differences between the genotypes or gene expression and prostate cancer survival. The association between rs4792800 and *TNFRSF13B* expression was assessed using meta-analysis to pool normalised effect size of the expression quantitative trait loci in 7893 samples from the GTEx database. Cell experimental results were represented as mean ± standard deviation and analysed via Student’s *t*-test. All analyses were conducted using Statistical Package for the Social Sciences software version 19.0.0 (IBM, Armonk, NY, USA), and two-sided *p* < 0.05 and *q* < 0.05 values were considered statistically significant.

## Results

The overall median follow-up time after radical prostatectomy was 54 months, at which time BCR was observed in 184 (40.2%) men (Table 1). A high pathological Gleason score, an advanced pathological stage, a positive surgical margin, and lymph node metastasis were found to be associated with an increased risk of BCR (*p* < 0.001).

**Table 1:** Clinicopathological characteristics of the 458 patients with prostate cancer after radical prostatectomy

Characteristics	*n* (%)	HR (95% CI)	*p*
Age at diagnosis, median (IQR)	66 (61–70)	1.02 (0.99–1.04)	0.149
PSA at diagnosis, median (IQR)	11.1 (7.1–17.5)	1.02 (1.01–1.02)	< 0.001
Pathologic Gleason score			
2–6	160 (35.3)	1.00	
7–10	293 (64.7)	2.19 (1.56–3.08)	< 0.001
Pathologic stage			
T1/T2	303 (67.2)	1.00	
T3/T4/N1	148 (32.8)	3.37 (2.51–4.52)	< 0.001
Surgical margin			
Negative	241 (72.6)	1.00	
Positive	91 (27.4)	2.80 (1.99–3.95)	< 0.001
Lymph node metastasis			
Negative	433 (95.6)	1.00	
Positive	20 (4.4)	13.4 (8.12–21.9)	< 0.001
Biochemical recurrence	184 (40.2)		
Median follow-up, months	54		

*HR* hazard ratio, *CI* confidence interval, *IQR* interquartile range, *PSA* prostate-specific antigen

**Fig. 1:**
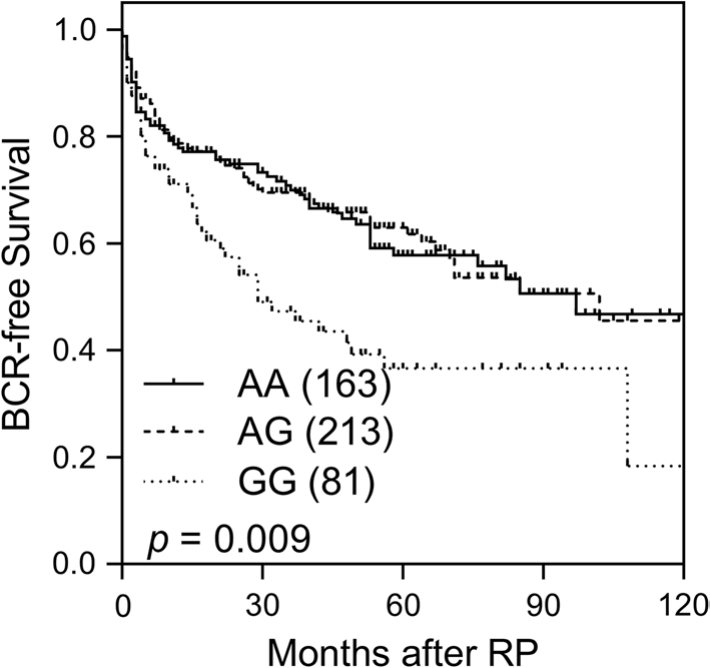
Kaplan–Meier survival analysis for biochemical recurrence-free survival based on *TNFRSF13B* rs4792800 genotypes. Numbers in parentheses indicate the number of patients. *BCR* biochemical recurrence, *RP* radical prostatectomy

Univariate associations between 19 SNPs in eight immunodeficiency pathway-related genes and BCR after radical prostatectomy are summarised in Table 2. After FDR correction, *TNFRSF13B* rs4792800 remained significantly associated with BCR in the recessive model (*q* = 0.019, Table 2). Homozygous carriers of the minor G allele of rs4792800 showed an increased risk of BCR (hazard ratio [HR] = 1.80, 95% confidence interval [CI] = 1.28–2.53, *p* = 0.001; Table 3; Fig. 1) when compared with the carriers of the major A allele. In the multivariate Cox model, after adjustment for clinicopathological predictors, *TNFRSF13B* rs4792800 was independently associated with BCR (HR = 1.78, 95% CI = 1.16–2.71, *p* = 0.008; Table 3), reinforcing the importance of *TNFRSF13B* rs4792800 in prostate cancer progression.

**Table 2:** Associations between immunodeficiency-related gene polymorphisms and biochemical recurrence

Gene	SNP ID	Chromosome	Position	MAF	HWE	Alleles	BCR					
							Additive		Dominant		Recessive	
							*p*	*q*	*p*	*q*	*p*	*q*
*CD8A*	rs1051386	2	86,865,584	0.182	0.382	A > G	0.626	0.317	0.438	0.274	‒	‒
*CD8A*	rs13023213	2	86,875,454	0.074	0.523	T > C	0.336	0.254	0.367	0.254	‒	‒
*ZAP70*	rs13034349	2	97,695,629	0.288	0.653	C > T	0.567	0.313	0.790	0.357	0.393	0.254
*ZAP70*	rs7565744	2	97,708,501	0.167	0.225	C > T	0.390	0.254	0.320	0.254	‒	‒
*ZAP70*	rs11686881	2	97,720,279	0.132	0.081	C > T	0.632	0.317	0.373	0.254	‒	‒
*ICOS*	rs11883722	2	204,509,090	0.435	1.000	G > A	0.347	0.254	0.246	0.254	0.761	0.352
*ICOS*	rs1559931	2	204,533,974	0.165	0.931	G > A	0.110	0.254	0.127	0.254	‒	‒
*ICOS*	rs4675379	2	204,534,340	0.128	0.988	G > C	0.031	0.120	0.049	0.159	‒	‒
*CD3E*	rs7928058	11	117,676,449	0.270	0.301	G > T	0.201	0.254	0.185	0.254	0.581	0.313
*CD3E*	rs7480736	11	117,677,574	0.470	0.896	C > T	0.114	0.254	0.136	0.254	0.275	0.254
*CD3E*	rs2231440	11	117,680,649	0.198	0.676	G > A	0.569	0.313	0.487	0.291	‒	‒
*CD3D*	rs3212264	11	117,721,444	0.445	0.810	C > A	0.171	0.254	0.385	0.254	0.164	0.254
*CIITA*	rs7196089	16	10,910,602	0.153	0.774	G > A	0.271	0.254	0.281	0.254	‒	‒
*CIITA*	rs11074939	16	10,919,210	0.326	0.289	G > A	0.852	0.376	0.996	0.411	0.702	0.332
*CIITA*	rs7404786	16	10,920,051	0.217	0.207	C > G	0.011	0.053	0.007	0.053	0.354	0.254
*CD19*	rs2070961	16	28,856,974	0.211	0.751	C > T	0.878	0.379	0.231	0.254	‒	‒
*TNFRSF13B*	rs4792800	17	16,785,892	0.410	0.522	A > G	0.009	0.053	0.258	0.254	0.001	**0.019**
*TNFRSF13B*	rs12938061	17	16,786,880	0.210	0.775	C > T	0.636	0.317	0.495	0.291	‒	‒
*TNFRSF13B*	rs4383187	17	16,788,634	0.278	0.855	C > T	0.923	0.389	0.698	0.332	0.331	0.254

*SNP* single nucleotide polymorphism, *BCR* biochemical recurrence, *MAF* minor alleles frequency, *HWE* Hardy-Weinberg equilibrium. ‒, not calculated due to insufficient numbers

**Table 3:** Associations between *TNFRSF13B* rs4792800 and biochemical recurrence

Genotype	*n*	BCR	5-year BFS	*p*	*q*	HR (95% CI)	*p*	HR (95% CI)^a^	*p* ^a^
AA	163	61	57.8			1.00		1.00	
AG	213	79	62.9			1.00 (0.72–1.40)	0.980	1.10 (0.72–1.67)	0.663
GG	81	44	36.6			1.80 (1.22–2.66)	0.003	1.88 (1.14–3.09)	0.013
AG/GG vs. AA				0.258	0.254	1.19 (0.88–1.62)	0.263	1.27 (0.86–1.87)	0.231
GG vs. AA/AG				0.001	0.019	1.80 (1.28–2.53)	0.001	1.78 (1.16–2.71)	0.008
Trend				0.009	0.053	1.31 (1.07–1.61)	0.010	1.35 (1.04–1.75)	0.022

*BCR* biochemical recurrence, *BFS* BCR-free survival, *HR* hazard ratio, *CI* confidence interval

^a^Adjustment for age, PSA at diagnosis, pathologic Gleason score, stage, surgical margin, and lymph node metastasis

**Fig. 2:**
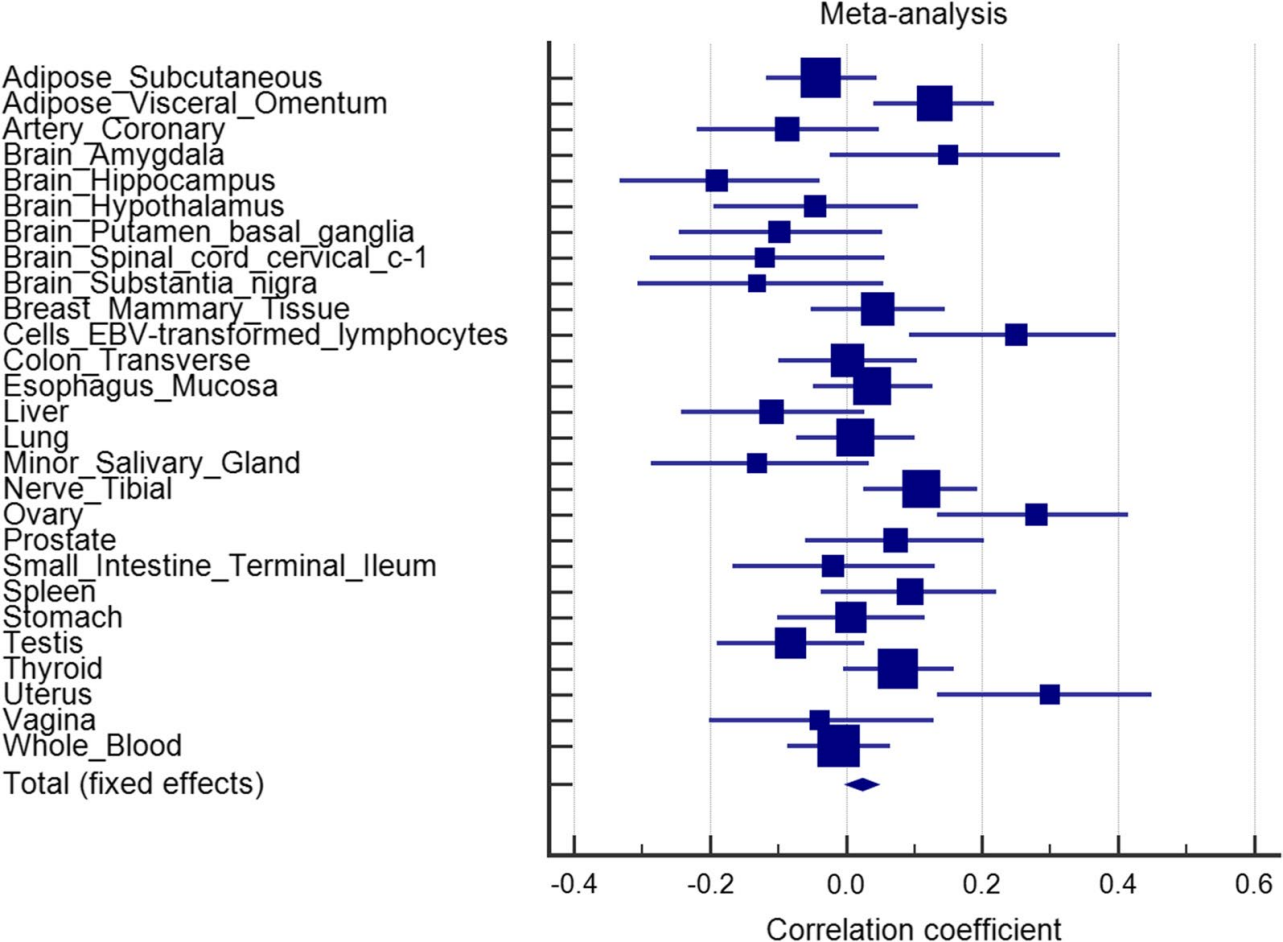
Meta-analysis of the correlation between rs4792800 and *TNFRSF13B* expression in 7893 tissue samples from the Genotype-Tissue Expression dataset

**Fig. 3:**
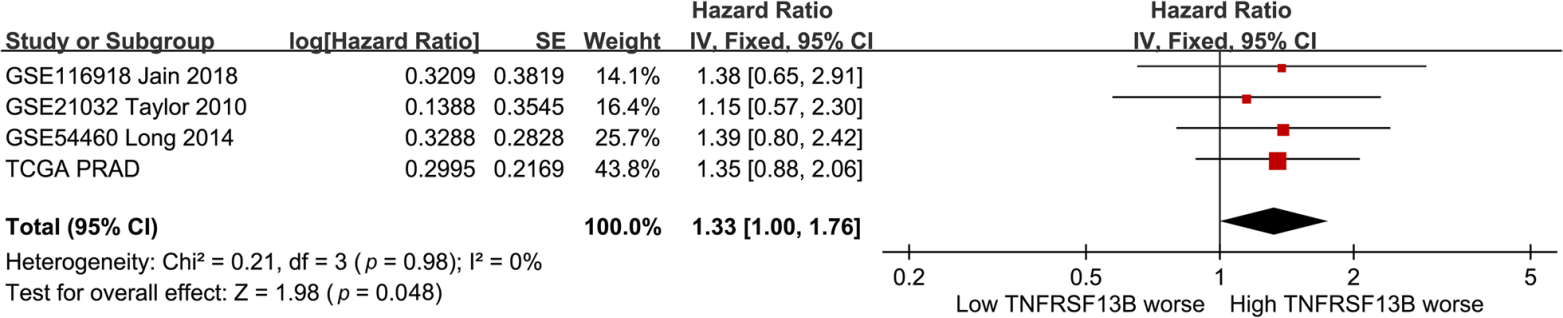
Meta-analysis of four studies evaluating the hazard ratio of high compared with low expression levels of *TNFRSF13B* for prostate cancer prognosis. *SE* standard error, *IV* inverse variance, *CI* confidence interval

*TNFRSF13B* rs4792800 and several linked (*r*^*2*^ > 0.8) proxy SNPs were found to locate in promoter/enhancer elements, overlapped with DNase I hypersensitive and RNA polymerase II binding regions in various cell types according to the HaploReg database (Additional file 1: Table S1), suggesting that rs4792800 might potentially regulate *TNFRSF13B* expression. Meta-analysis of 7893 samples across 27 types of human tissues showed increased *TNFRSF13B* expression for rs4792800 A > G transition in the GTEx data (*p* = 0.038, Fig. 2). Further, a meta-analysis was performed using four independent cohorts of 991 prostate cancer patients to evaluate the prognostic significance of *TNFRSF13B*. Patients in the datasets were divided into high and low expression groups according to the median expression levels of *TNFRSF13B*, and differences in their five-year survival were compared. Patients in the high *TNFRSF13B* expression group showed poorer prognosis than those in the low expression group (HR = 1.33, 95% CI = 1.00–1.76, *p* = 0.048; Fig. 3). These findings imply that the upregulated *TNFRSF13B* expression in patients carrying rs4792800 G allele predisposes the patient to an increased risk of BCR.

**Fig. 4:**
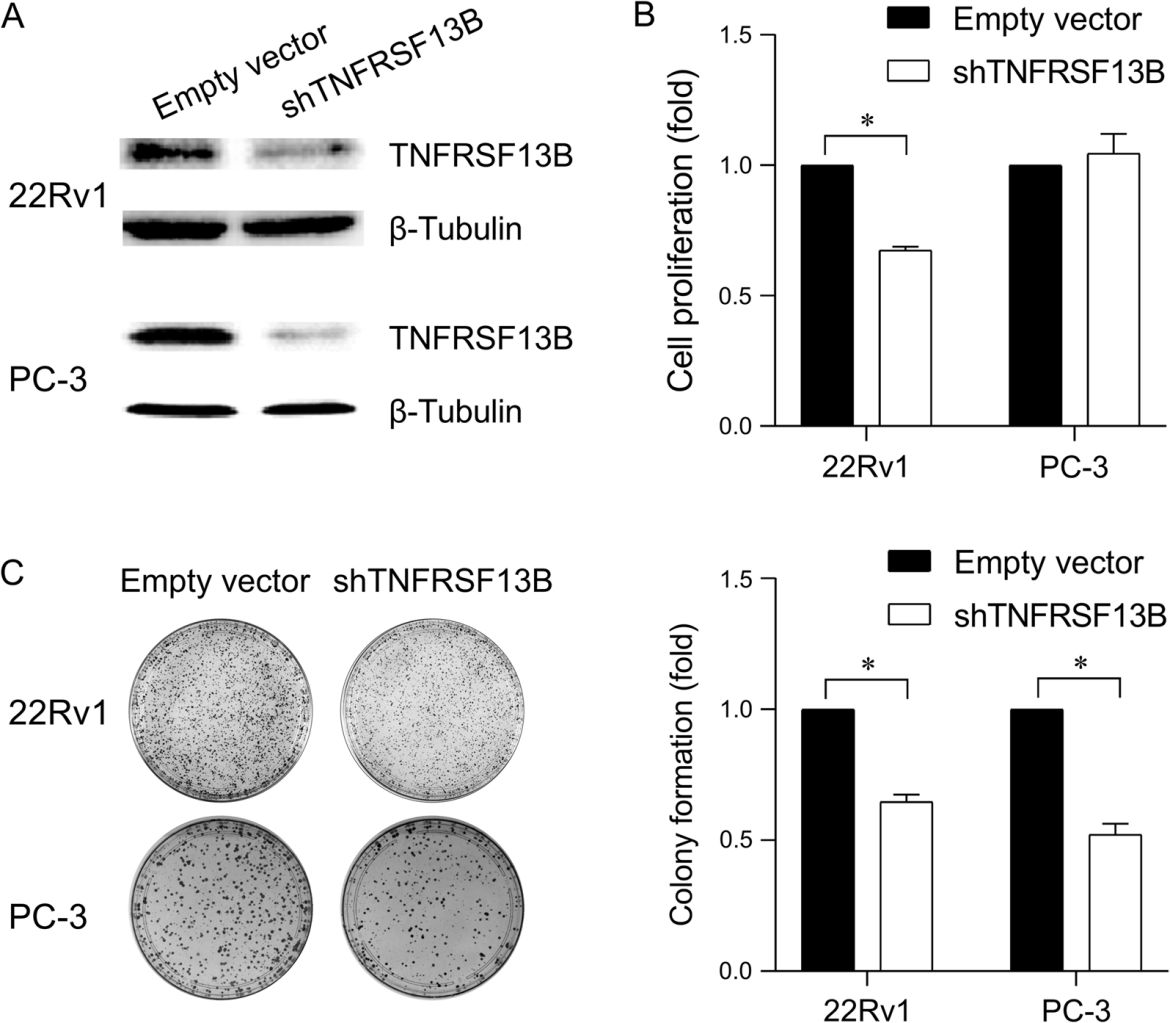
Silencing *TNFRSF13B* expression decreases the colony formation potential of human prostate cancer cells. **A** Silencing *TNFRSF13B* expression with short hairpin RNAs (shRNAs) decreased protein expression in the human prostate cancer 22Rv1 and PC-3 cell lines. Cells were transfected with an empty vector or *TNFRSF13B* shRNAs (shTNFRSF13B) using lentivirus, and TNFRSF13B protein expression in cells was examined via western blotting. **B** *TNFRSF13B* knockdown decreases the proliferation of 22Rv1 cells but not of PC-3 cells. Cells stably expressing an empty vector or shTNFRSF13B were seeded in 96-well plates and allowed to proliferate for four days. Cell proliferation was then estimated using MTT assay. **C** *TNFRSF13B* knockdown decreases the colony formation potential of both 22Rv1 and PC-3 cells. Cells stably expressing an empty vector or shTNFRSF13B were seeded in 6-well plates and allowed to grow for three weeks. The colonies were fixed and counted using the ImageJ software. Data are represented as mean ± standard deviation values from three independent experiments. *p < 0.05

To understand the function of TNFRSF13B in prostate cancer progression, *TNFRSF13B* was knocked down in 22Rv1 and PC-3 human prostate cancer cell lines. The expression of the TNFRSF13B protein was notably downregulated in both 22Rv1 and PC-3 cells owing to the introduction of shRNAs targeting *TNFRSF13B* (shTNFRSF13B) when compared with cells transfected with an empty vector (Fig. 4A; Additional file 2). Cell proliferation was assessed using MTT assay, and the growth rate of *TNFRSF13B*-silenced 22Rv1 cells was found to be significantly lower than that of an empty vector control (Fig. 4B). However, the cell growth was not affected by *TNFRSF13B* knockdown in PC-3 cells. Additionally, the colony formation assay was performed which showed the tumour-initiating capabilities of cells. The colony forming ability of *TNFRSF13B*-silenced 22Rv1 cells was decreased by 35.3% when compared with 22Rv1 cells stably expressing the vector control, whereas the colony forming ability of *TNFRSF13B*-silenced PC-3 cells was decreased by 47.8% when compared with PC-3 cells stably expressing the vector control (both *p* < 0.05, Fig. 4C). Collectively, these results suggest that silencing *TNFRSF13B* might inhibit tumorigenesis through modulating prostate cancer cell colony formation.

**Fig. 5:**
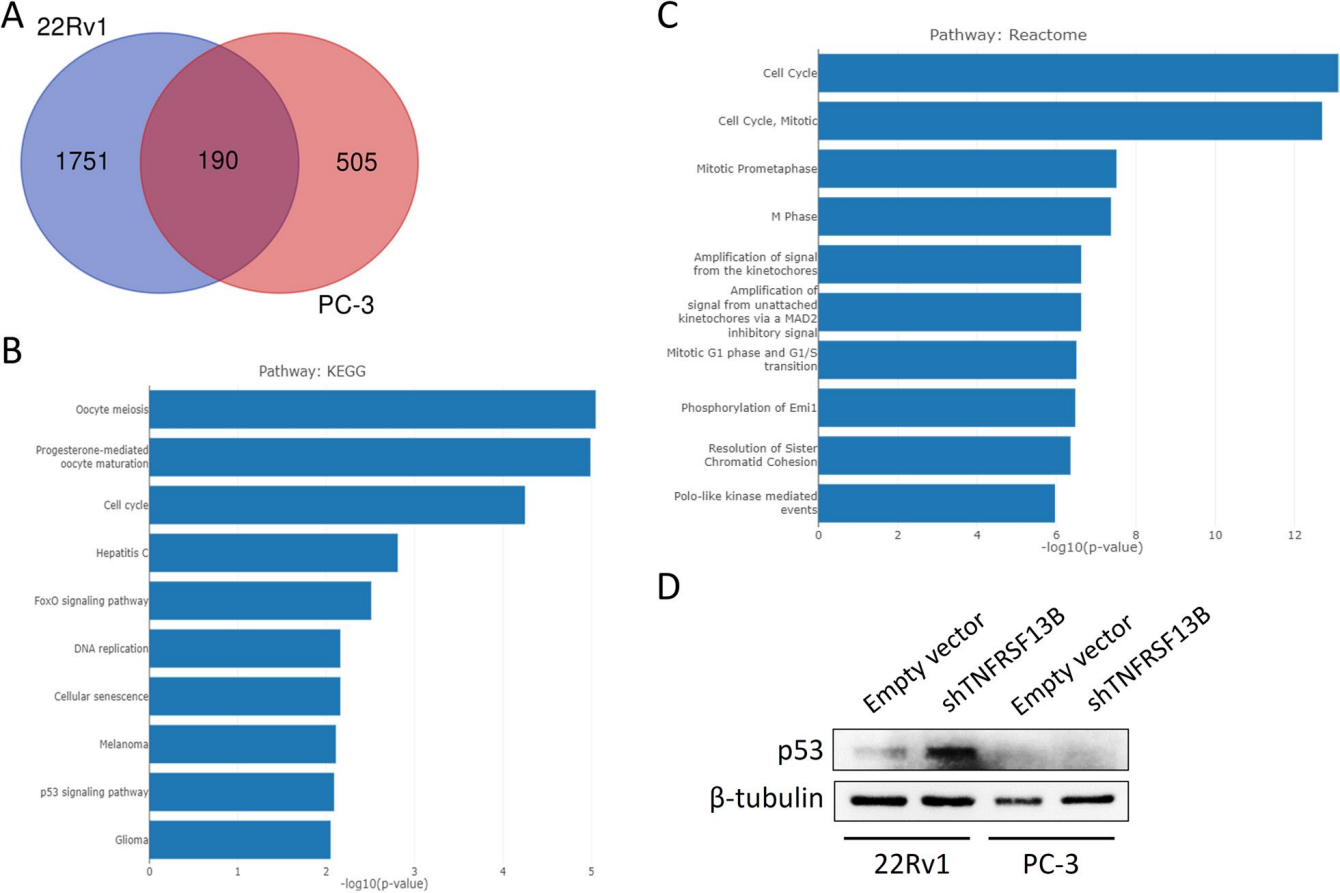
Gene expression profiling identified that cell cycle-related pathways are altered after *TNFRSF13B* short hairpin RNA (shTNFRSF13B) transfection in human prostate cancer cells. **A** Venn diagram analysis of all significantly differentially expressed (SDE) genes in 22Rv1 and PC-3 cells, displayed as the number of genes. **B** KEGG pathway analysis of 190 common SDE genes expressed in both 22Rv1 and PC-3 cells. **C** Reactome pathway analysis of 190 common SDE genes expressed in both 22Rv1 and PC-3 cells. **D** Knockdown of *TNFRSF13B* resulted in an increase of p53 protein expression in 22Rv1 cells, but no p53 expression was observed in PC-3 cells. Cells were transfected with an empty vector or shTNFRSF13B, and p53 protein expression was examined via western blotting. The representative western blot was obtained from three independent experiments

To further elucidate the molecular mechanisms underlying the effects of *TNFRSF13B* in regulating the growth of prostate cancer cells, the differentially expressed genes between control- and shTNFRSF13B-transfected PC-3 and 22Rv1 cells were analysed by microarray gene expression profiling. A total of 695 and 1941 SDE genes were identified in *TNFRSF13B*-silenced PC-3 and *TNFRSF13B*-silenced 22Rv1 cells, respectively. Venn diagram analysis showed that 190 SDE genes were commonly dysregulated upon *TNFRSF13B* knockdown in both cell lines (Fig. 5A; Additional file 1: Table S2). This list of 190 common SDE genes was used for further enrichment and biological process annotation based on the KEGG and Reactome pathway databases. The most strongly related pathways to the genetic changes induced by *TNFRSF13B* inhibition were the meiosis, cell cycle, FoxO, DNA replication, senescence, and p53 signalling pathways in KEGG enrichment analysis (Fig. 5B). Similarly, the cell cycle pathway also had the highest score in the Reactome analysis (*p* = 10^− 13.1^, Fig. 5C). These enrichment results are relevant because the cell cycle and p53 signalling pathways are known to contribute to the aggressive nature of cancers. These results further confirmed the important role of *TNFRSF13B* as a contributor to prostate cancer cell proliferation and colony formation.

Since p53 is expressed in 22Rv1 cells but is not expressed in PC-3 cells [35], the expression of p53 protein was assessed by western blot analysis. Knockdown of *TNFRSF13B* increased the expression level of p53 in 22Rv1 cells compared with that in the cells transfected with the empty control vector; however, no p53 expression was observed in the PC-3 cells (Fig. 5D; Additional file 2). Furthermore, gene expression profiling revealed that silencing *TNFRSF13B* had the opposite effect on several cell cycle and p53 signalling pathway genes between p53-expressing 22Rv1 cells and p53-null PC-3 cells. For example, cyclin B1 and several cell division cycle genes were upregulated in 22Rv1 cells but downregulated in PC-3 cells (Additional file 1: Table S3). These differentially expressed genes may partly explain why silencing *TNFRSF13B* expression caused significant growth inhibition in 22Rv1 cells, but not in PC-3 cells.

## Discussion

In this pilot study, 19 htSNPs in eight immunodeficiency pathway-related genes were screened and the effects of these variants on BCR after radical prostatectomy for prostate cancer were investigated. *TNFRSF13B* rs4792800 was found to be significantly associated with BCR by multivariate analysis and multiple comparisons. In addition, rs4792800 affected *TNFRSF13B* expression, which was correlated with patient prognosis. Further investigations revealed that silencing *TNFRSF13B* gene reduced colony formation in two prostate cancer cell lines, suggesting a possible role for *TNFRSF13B* in prostate cancer pathogenesis.

TNFRSF13B, also known as transmembrane activator and calcium modulator and cyclophilin ligand interactor (TACI), is a member of the tumour necrosis factor (TNF) receptor superfamily. TNFRSF13B is the main receptor of the TNF superfamily member 13 (TNFSF13, also known as a proliferation-inducing ligand) and 13B (also known as B-cell activating factor). Upon TNFSF13/13B binding to TNFRSF13B, the complex recruits TNF receptor-associated factors 2 and 6 [36], activates the nuclear factor kappa B signalling pathway, and affects multiple events in immunomodulation such as immunoglobulin recombination and B cell activation, proliferation, and survival [37]. Abnormal TNFRSF13B signalling has been related to autoimmune disorders [38]. Approximately 10% of patients with common variable immunodeficiency carry mutations in the *TNFRSF13B* [39, 40], and *TNFRSF13B*-knockout mice display symptoms of systemic lupus erythematosus-like autoimmune diseases [41]. It has been suggested that patients with defective immunity are at an increased risk of developing cancer. The soluble form of TNFRSF13B can be detected in blood and is found to be elevated in patients with chronic lymphocytic leukaemia [42]. Moreover, TNFSF13 is considered as an anti-apoptotic cytokine because its overexpression is frequently correlated with cancer progression [43–45] and it protects tumour cells from apoptosis by promoting cell cycle progression and cell proliferation in many cancer types [46]. A recombinant fusion protein of the extracellular domain of TNFRSF13B and the human IgG1-Fc (TACI-Ig) inhibited TNFRSF13B signalling and induced apoptosis of myeloma cells in vitro [47], and treatment using TACI-Ig was associated with some anti-tumour activities in multiple myeloma and Waldenström’s macroglobulinemia in a phase I/II trial [48]. In this study, *TNFRSF13B* rs4792800, an intronic variant, was found to be a significant variant associated with prostate cancer recurrence. Using functional annotation, a 3’-untranslated region proxy variant rs55701306 was identified in high linkage disequilibrium (*r*^*2*^ = 0.96) with rs4792800 as a strong expression quantitative trait locus for *TNFRSF13B* in human lymphoblastoid cells [49]. To date, several studies have reported significant associations between genetic polymorphisms in *TNFRSF13B* and the risks of several types of cancer. A combined analysis of African and European ancestry populations identified a missense variant (rs34562254) in *TNFRSF13B* associated with multiple myeloma risk [50]. It has also been reported that several genetic variants in *TNFRSF13B* influence gene expression and susceptibility to chronic lymphocytic leukaemia [51]. Furthermore, a significant association of *TNFRSF13B* rs7501462 with survival was identified in a large cohort of 10,084 patients with invasive epithelial ovarian cancer [52]. Although the link between *TNFRSF13B* expression and prostate cancer remains unknown, gene expression analysis based on public datasets showed that *TNFRSF13B* overexpression correlated with poor prognosis in patients with prostate cancer. A recent study demonstrated that silencing *TNFRSF13B* led to a significantly increased death in breast cancer cells through inhibiting anti-apoptotic/pro-survival mediators, TNF receptor superfamily member 1B, BCL2 apoptosis regulator, and RELA proto-oncogene NF-KB subunit, along with cell cycle arrest through inducing cyclin D2 and proliferating cell nuclear antigen [53]. In line with our study, *TNFRSF13B* knockdown significantly decreased the colony growth of 22Rv1 and PC-3 human prostate cancer cells, suggesting the potential role of *TNFRSF13B* in prostate cancer progression. The results of our transcriptomic analysis indicated that *TNFRSF13B* knockdown significantly modulates the cell cycle and p53 signalling pathways, which are two critical pathways in regulating cancer cell growth [54]. Silencing *TNFRSF13B* increased the expression of p53 and suppressed p53-expressing 22Rv1 cell growth, whereas cell proliferation was not affected in p53-null PC-3 cells, suggesting that p53 status might be associated with *TNFRSF13B*-driven prostate cancer cell proliferation [55].

Although genetic studies from our and other groups have identified multiple prognostic genes associated with prostate cancer progression, including aldehyde oxidase 1 [56], solute carrier family 35 member B4 [20], and carboxylesterase 1 [57]; however, to our knowledge, this is the first study to link *TNFRSF13B* to prostate cancer. Nevertheless, several limitations of the present study need to be considered. First, the sample size was relatively small to detect moderately minor risks. Second, this is a hospital-based study and the participants may not represent the general population. Third, only htSNPs of key genes involved in the immunodeficiency pathway were examined, therefore, more genetic variants at other loci should be tested. Fourth, the median follow-up time of this study was only 54 months, therefore, studies with longer follow-up time should be conducted to validate our results in other gold standard endpoints, such as overall survival or prostate cancer-specific survival. Fifth, functional significance of rs4792800 and *TNFRSF13B* expression was largely from bioinformatic analyses, and thus need to be confirmed in future clinical and biological studies. Finally, the current study was carried out in the Chinese population; therefore, similar associations among different populations should be further investigated.

## Conclusions

**Fig. 6:**
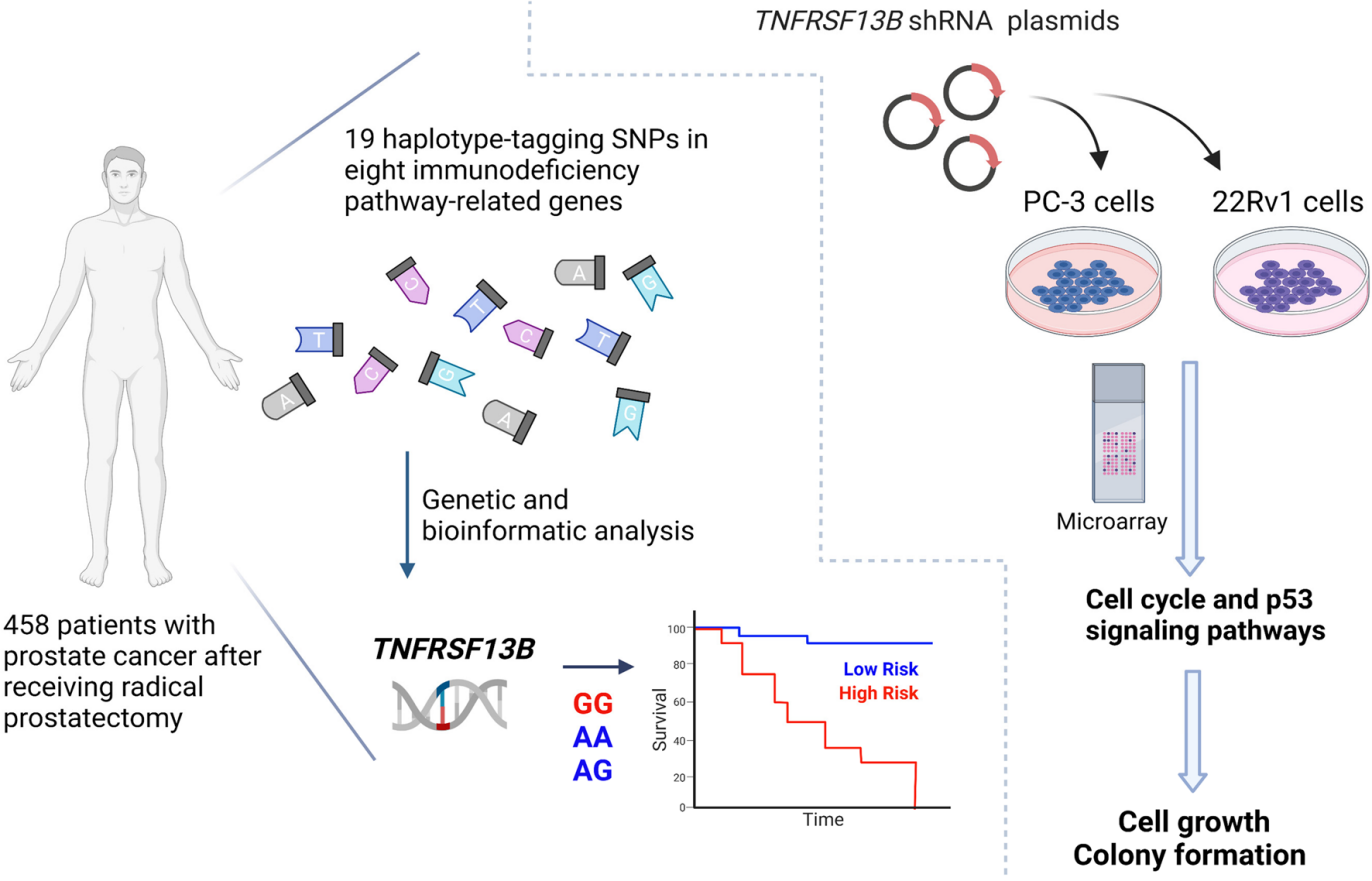
Genetic and functional analyses identify the role of *TNFRSF13B* in prostate cancer progression. Homozygous carriers of the minor G allele of *TNFRSF13B* rs4792800 were identified to be significantly associated with an increased risk of biochemical recurrence in 458 patients with prostate cancer after receiving radical prostatectomy. Further gene expression profiling revealed that silencing *TNFRSF13B* can inhibit prostate cancer cell growth and colony formation through modulating the cell cycle and p53 signalling pathways

This study provides evidence that genetic variants of immunodeficiency pathway-related genes, especially *TNFRSF13B*, contribute to prostate cancer recurrence through modulating the cell cycle and p53 signalling pathways (Fig. 6). These findings suggest that *TNFRSF13B* might be a novel prognostic biomarker and potential therapeutic target in prostate cancer. However, larger, well-designed studies with diverse populations and functional evaluations should be conducted to validate our findings.

## Supplementary Information

**Additional file:  Supplementary Tables. Table S1.** Regulatory annotation of *TNFRSF13B* rs4792800 and its linked variants. **Table S2**. List of differentially expressed genes upon *TNFRSF13B* knockdown that overlapped in both 22Rv1 and PC-3 human prostate cancer cells. **Table S3**. List of differentially expressed cell cycle- and p53 signalling pathway-related genes with opposite responses to *TNFRSF13B* knockdown between 22Rv1 and PC-3 cells.

**Additional file 2:** The images of the untrimmed western blots.

## Data Availability

The data presented in this study are available on request from the corresponding author.

## Abbreviations

PSA: Prostate-specific antigen
BCR: Biochemical recurrence
TNFRSF13B: Tumour necrosis factor receptor superfamily member 13B
htSNP: Haplotype-tagging single-nucleotide polymorphism
GTEx: Genotype-tissue expression
TCGA: The Cancer Genome Atlas
shRNA: Short hairpin RNA
PBS: Phosphate-buffered saline
MTT: 3-(4,5-dimethylthiazol-2-yl)-2,5 diphenyltetrazolium bromide
FDR: False discovery rate
HR: Hazard ratio
CI: Confidence interval
TACI: Transmembrane activator and calcium modulator and cyclophilin ligand interactor
TNF: Tumour necrosis factor
TNFSF13: Tumour necrosis factor superfamily members 13
