# Supplementary material for: TNFRSF13B is a potential contributor to prostate cancer
Source: Cancer Cell Int. 2022 May 6;22:180. doi: 10.1186/s12935-022-02590-2 (PMC9074181; doi:10.1186/s12935-022-02590-2)
Supplement: Supplementary file 1 — Additional file: Supplementary Tables. Table S1. Regulatory annotation of TNFRSF13B rs4792800 and its linked variants. Table S2. List of differentially expressed genes upon TNFRSF13B knockdown that overlapped in both 22Rv1 and PC-3 human prostate cancer cells. Table S3. List of differentially expressed cell cycle- and p53 signalling pathway-related genes with opposite responses to TNFRSF13B knockdown between 22Rv1 and PC-3 cells. [file 12935_2022_2590_MOESM1_ESM.doc]

**Table S1** Regulatory annotation of *TNFRSF13B* rs4792800 and its linked variants.

| Chromosome | Position | LD (r²) | SNP ID | Reference allele | Alternate allele | ASN frequency | Promoter histone marks | Enhancer histone marks | DNAse | Proteins bound | Motifs changed | dbSNP function annotation |
| --- | --- | --- | --- | --- | --- | --- | --- | --- | --- | --- | --- | --- |
| 17 | 16936468 | 0.82 | rs74998556 | A | T | 0.36 |  | IPSC |  |  | Foxp1, RREB-1 |  |
| 17 | 16937297 | 0.91 | rs11871721 | C | G | 0.39 |  | IPSC |  |  | AIRE |  |
| 17 | 16937548 | 0.93 | rs4792798 | G | A | 0.38 |  | IPSC, BLD | BLD | POL2 | DMRT4 |  |
| 17 | 16937682 | 0.93 | rs4792799 | G | A | 0.38 |  | IPSC, BLD | IPSC, MUS | POL2 | BDP1, EWSR1-FLI1, Pax-5 |  |
| 17 | 16938999 | 0.95 | rs55672385 | A | C | 0.38 | BLD | ESDR, ESC, IPSC, BRN, MUS, THYM | THYM, LNG |  | Hic1, Myc, Rad21, Roaz |  |
| 17 | 16939133 | 0.96 | rs55701306 | C | T | 0.39 |  | ESDR, ESC, IPSC, BRN, MUS, THYM, BLD | ESDR, BLD, BLD, BLD, MUS, THYM, BLD, MUS | POL24H8 | BHLHE40, Nkx2, PLAG1, PPAR, Sin3Ak-20 | 3'-UTR |
| 17 | 16939677 | 0.96 | rs34562254 | G | A | 0.39 |  | ESC, ESDR, IPSC, THYM, BLD |  | POL2 | CCNT2, Ets, Glis2, Pax-5, RXRA, ZBTB7A, ZNF219 | missense |
| 17 | 16941853 | 1 | **rs4792800** | A | G | 0.39 |  | FAT, STRM, BRN, MUS | SKIN, SKIN, HRT, MUS, MUS, GI, MUS, MUS, BRN, SKIN, LNG |  | Cdx, Cphx, Dbx2, HEY1, Hdx, Hoxb4, Isl2, Nkx6-1, Pdx1, Pou1f1, Pou4f3, TATA | intronic |
| 17 | 16945251 | 0.94 | rs4500785 | C | G | 0.38 |  | IPSC, BLD, SKIN, BRN, MUS, PANC, LIV | IPSC | POL2 | AP-2, NRSF | intronic |
| 17 | 16945436 | 0.96 | rs4561508 | C | T | 0.38 |  | ESC, IPSC, BLD, BRN, MUS, PANC, LIV |  |  | GLI | intronic |
| 17 | 16948136 | 0.93 | rs57382045 | G | A | 0.38 |  | BLD, BRN | BLD |  | Brachyury, Msx-1 | intronic |
| 17 | 16951166 | 0.91 | rs74892229 | G | A | 0.38 |  |  | BLD |  | ERalpha-a, Esr2, Ets, NRSF | intronic |
| 17 | 16953548 | 0.91 | rs57293260 | A | T | 0.38 |  | BLD, THYM | BLD, BLD, BLD, LNG, BLD | CTCF | Arid5a | intronic |
| 17 | 16953863 | 0.9 | rs57166795 | G | A | 0.38 |  | BLD, BRN, THYM |  |  | GLI, HNF4, Myf, NRSF, RXRA, VDR, Znf143 | intronic |
| 17 | 16960324 | 0.91 | rs4985726 | C | G | 0.38 |  |  | BLD |  |  | intronic |

**Table S2** List of differentially expressed genes upon *TNFRSF13B* knockdown that overlapped in both 22Rv1 and PC-3 human prostate cancer cells.

| Gene name | Gene ID | Fold change in 22Rv1 cells (log2 ratio) | Fold change in PC-3 cells (log2 ratio) |
| --- | --- | --- | --- |
| *AASS* | 10157 | 3.09 | 3.07 |
| *ADAMTS1* | 9510 | -3.15 | 2.42 |
| *ADGRG1* | 9289 | -3.87 | -2.61 |
| *AFF3* | 3899 | 3.15 | 2.65 |
| *AGR2* | 10551 | 2.16 | 4.18 |
| *ANKRD29* | 147463 | 3.83 | 3.18 |
| *ARG2* | 384 | -2.26 | 2.69 |
| *ARHGAP11B* | 89839 | 3.41 | -2.02 |
| *ARHGEF38* | 54848 | 2.04 | 3.69 |
| *ARRDC3* | 57561 | -5.28 | 4.51 |
| *ASNS* | 440 | -2.20 | -2.45 |
| *ASPM* | 259266 | 3.00 | -2.55 |
| *ATP13A3* | 79572 | 2.76 | -2.01 |
| *ATP8A1* | 10396 | -2.18 | 2.35 |
| *ATP8B1* | 5205 | 4.04 | 2.29 |
| *AURKB* | 9212 | 2.95 | -2.37 |
| *AXIN2* | 8313 | -2.08 | 2.21 |
| *BIRC3* | 330 | -8.35 | 2.91 |
| *BMP4* | 652 | -2.46 | 3.24 |
| *BST2* | 684 | -2.47 | 17.93 |
| *BUB1* | 699 | 2.29 | -2.14 |
| *CA13* | 377677 | -2.46 | 2.58 |
| *CADM2* | 253559 | -3.07 | 2.60 |
| *CAPN3* | 825 | 2.80 | 2.88 |
| *CCDC18* | 343099 | 2.43 | -2.09 |
| *CCNB1* | 891 | 2.31 | -2.13 |
| *CDC20* | 991 | 2.91 | -2.58 |
| *CDC25C* | 995 | 2.01 | -2.57 |
| *CDC45* | 8318 | 4.25 | -2.56 |
| *CDCA2* | 157313 | 2.88 | -2.19 |
| *CDCA3* | 83461 | 2.85 | -3.46 |
| *CDCA8* | 55143 | 2.08 | -2.76 |
| *CDH6* | 1004 | 2.03 | 2.99 |
| *CENPA* | 1058 | 2.72 | -2.04 |
| *CENPE* | 1062 | 3.50 | -2.28 |
| *CENPF* | 1063 | 2.24 | -2.73 |
| *CENPI* | 2491 | 2.99 | -2.42 |
| *CEP55* | 55165 | 2.73 | -2.32 |
| *CEP78* | 84131 | 2.37 | -2.19 |
| *CHAC1* | 79094 | -2.18 | -3.43 |
| *CHAF1B* | 8208 | 2.03 | -2.24 |
| *CLDN1* | 9076 | -4.22 | 4.81 |
| *CLDN11* | 5010 | -5.63 | -3.47 |
| *CLSPN* | 63967 | 2.68 | -2.15 |
| *CORO2A* | 7464 | -2.10 | 2.05 |
| *CPS1* | 1373 | -2.76 | -2.02 |
| *CSF1* | 1435 | 3.50 | 4.16 |
| *DDIAS* | 220042 | 2.00 | -2.02 |
| *DGCR8* | 54487 | 3.32 | -2.73 |
| *DMGDH* | 29958 | -3.84 | 2.86 |
| *DNAH7* | 56171 | -2.13 | 4.38 |
| *DNAJB4* | 11080 | -3.15 | 2.95 |
| *DNAJB9* | 4189 | -2.65 | 2.46 |
| *DTL* | 51514 | 2.02 | -2.14 |
| *DTX3L* | 151636 | 2.94 | 4.87 |
| *DUSP10* | 11221 | -4.83 | 2.40 |
| *E2F1* | 1869 | 2.39 | -2.06 |
| *E2F3* | 1871 | 2.24 | -2.24 |
| *E2F7* | 144455 | 2.89 | -2.00 |
| *EDN1* | 1906 | -4.87 | 2.02 |
| *ELF1* | 1997 | 3.06 | 2.50 |
| *EPHA4* | 2043 | -2.82 | 2.39 |
| *ERAP1* | 51752 | -3.43 | 2.45 |
| *FAM46A* | 55603 | -7.56 | 2.88 |
| *FAM83D* | 8161 | 2.75 | -2.08 |
| *FANCD2* | 2177 | 2.54 | -3.39 |
| *FANCI* | 55215 | 2.26 | -2.44 |
| *FBXO43* | 286151 | 3.78 | -2.53 |
| *FBXO5* | 26271 | 2.43 | -2.54 |
| *FERMT1* | 55612 | 2.55 | -2.30 |
| *GABARAPL1* | 2371 | -20.77 | 2.90 |
| *GLYATL2* | 219970 | -5.77 | 2.63 |
| *GMPR* | 2766 | 2.67 | 2.34 |
| *GNAI1* | 2770 | -3.79 | 2.03 |
| *GPCPD1* | 56261 | 2.57 | 2.04 |
| *GPR155* | 151556 | -2.09 | 2.94 |
| *HAS2* | 3037 | -7.50 | 3.44 |
| *HERC5* | 51191 | 3.67 | 7.45 |
| *HIST1H1C* | 3006 | 2.41 | -2.04 |
| *HIST1H1E* | 3008 | 2.77 | -3.54 |
| *HIST1H2AB* | 8335 | 2.68 | -2.69 |
| *HIST1H3B* | 8358 | 3.87 | -4.59 |
| *HIST2H3A* | 333932 | 2.06 | -2.88 |
| *HMMR* | 3161 | 2.53 | -2.06 |
| *HOXB-AS3* | 404266 | 3.44 | 2.08 |
| *HPGD* | 3248 | 2.29 | 5.23 |
| *IGF1R* | 3480 | 2.62 | 2.40 |
| *IKZF2* | 22807 | 2.73 | 2.53 |
| *KCNMA1* | 3778 | 3.70 | -2.02 |
| *KCNQ5* | 56479 | 2.22 | -2.08 |
| *KIF14* | 9928 | 3.57 | -2.43 |
| *KIF15* | 56992 | 2.31 | -2.62 |
| *KIF20A* | 10112 | 3.28 | -2.39 |
| *KIF22* | 3835 | 2.17 | -2.33 |
| *KIFC1* | 3833 | 3.11 | -2.72 |
| *KLF7* | 8609 | -3.71 | 2.36 |
| *LAMP3* | 27074 | -8.60 | 2.76 |
| *LCP1* | 3936 | 2.30 | 2.73 |
| *LGR5* | 8549 | -3.50 | 2.43 |
| *LIPH* | 200879 | -5.27 | 8.24 |
| *LPCAT1* | 79888 | -3.26 | 2.01 |
| *LRRC34* | 151827 | 2.40 | -2.28 |
| *MCEE* | 84693 | 2.17 | 2.08 |
| *MEF2C* | 4208 | -4.10 | 6.92 |
| *MEST* | 4232 | 2.35 | -2.38 |
| *MIS18A* | 54069 | 2.03 | -2.02 |
| *MKI67* | 4288 | 2.05 | -2.24 |
| *MMP1* | 4312 | -3.97 | 3.20 |
| *MND1* | 84057 | 3.76 | -2.03 |
| *MNS1* | 55329 | 3.78 | -2.41 |
| *MTHFD1L* | 25902 | 2.70 | -2.12 |
| *MX2* | 4600 | -2.35 | 16.90 |
| *MYBL2* | 4605 | 2.67 | -2.47 |
| *NCAPG* | 64151 | 2.71 | -2.88 |
| *NCAPH* | 23397 | 2.04 | -2.65 |
| *NLGN1* | 22871 | 2.59 | 2.19 |
| *NMI* | 9111 | 2.67 | 4.30 |
| *NMRK1* | 54981 | -2.05 | 2.20 |
| *NPR3* | 4883 | 2.71 | 2.17 |
| *NUSAP1* | 5120 | 2.25 | -2.66 |
| *OAS3* | 4940 | 2.00 | 6.65 |
| *PARP14* | 54625 | 3.79 | 3.37 |
| *PARP9* | 83666 | 2.71 | 7.44 |
| *PARPBP* | 55010 | 2.12 | -2.16 |
| *PDE4B* | 5142 | -5.99 | 2.02 |
| *PDK4* | 5166 | -10.59 | 2.22 |
| *PLBD1* | 79887 | 2.85 | -2.42 |
| *PLK1* | 5347 | 2.14 | -2.41 |
| *PLK4* | 10733 | 2.18 | -2.70 |
| *PLSCR1* | 5359 | 4.39 | 4.37 |
| *PLSCR2* | 57047 | 2.79 | 2.40 |
| *POLA1* | 5422 | 3.49 | -2.24 |
| *PPEF1* | 5475 | -2.08 | 2.02 |
| *PRIM1* | 5557 | 2.22 | -2.75 |
| *PRIM2* | 5558 | 3.23 | -2.23 |
| *PRR11* | 55771 | 2.72 | -2.11 |
| *PSMB9* | 5698 | 2.46 | 10.28 |
| *PSMC3IP* | 29893 | 2.69 | -2.32 |
| *PTEN* | 5728 | -2.98 | 2.26 |
| *PTGFR* | 5737 | 2.60 | -2.84 |
| *QPCT* | 25797 | -4.19 | 2.35 |
| *RAD51AP1* | 10635 | 2.46 | -3.12 |
| *RAD54L* | 8438 | 4.91 | -2.03 |
| *RANBP1* | 5902 | 2.40 | -2.05 |
| *RGCC* | 28984 | -6.82 | 8.27 |
| *RHOBTB1* | 9886 | -4.54 | 2.18 |
| *RPL5* | 6125 | 2.19 | -2.03 |
| *RPS6KA1* | 6195 | -2.57 | -2.25 |
| *RRM2* | 6241 | 2.14 | -2.34 |
| *SAT1* | 6303 | -4.41 | 3.95 |
| *SATB1* | 6304 | -2.54 | 4.08 |
| *SCIN* | 85477 | -2.03 | 2.54 |
| *SEMA6D* | 80031 | -3.21 | 2.99 |
| *SERPINI1* | 5274 | -3.24 | 2.81 |
| *SESN2* | 25788 | 2.23 | -2.03 |
| *SESN2* | 83667 | -2.23 | -2.03 |
| *SLC16A9* | 220963 | -2.13 | 2.58 |
| *SLC1A4* | 6509 | -2.31 | -2.64 |
| *SLC43A3* | 29015 | 2.08 | -2.19 |
| *SLC6A16* | 28968 | 2.63 | 2.58 |
| *SLC7A11* | 23657 | -3.21 | -2.58 |
| *SLC7A5* | 8140 | -3.60 | -2.57 |
| *SMIM14* | 201895 | -2.03 | 2.02 |
| *SNX5* | 27131 | 7.26 | -2.32 |
| *SP110* | 3431 | -2.08 | 8.35 |
| *SPC25* | 57405 | 3.77 | -2.14 |
| *SPTSSB* | 165 | -2.40 | 5.05 |
| *SQSTM1* | 8878 | 4.01 | 2.09 |
| *STEAP4* | 79689 | -4.24 | 3.25 |
| *STX11* | 8676 | -2.91 | 2.05 |
| *SUV39H2* | 79723 | 2.05 | -2.44 |
| *TCP11L2* | 255394 | -19.26 | 2.62 |
| *TLR3* | 7098 | 3.80 | 8.44 |
| *TMEM150C* | 441027 | 3.85 | 4.62 |
| *TMEM27* | 57393 | -2.71 | 4.58 |
| *TNC* | 3371 | -3.12 | -2.49 |
| *TNFSF4* | 7292 | -2.30 | 2.18 |
| *TNNI3K* | 51086 | -2.04 | 2.22 |
| *TRIB3* | 57761 | -2.88 | -2.87 |
| *TRIM38* | 10475 | -4.40 | 2.70 |
| *TRMU* | 55687 | 2.00 | -2.03 |
| *TROAP* | 10024 | 2.27 | -2.01 |
| *TSPAN8* | 7103 | -2.58 | 5.15 |
| *TTC6* | 319089 | 2.12 | 5.28 |
| *TYMS* | 7298 | 3.83 | -2.02 |
| *WDR76* | 79968 | 2.71 | -2.21 |
| *YPEL5* | 51646 | -2.59 | 2.03 |
| *ZFP36* | 7538 | -4.27 | 2.66 |
| *ZGRF1* | 55345 | 2.43 | -2.09 |
| *ZNF704* | 619279 | 3.28 | 2.39 |

**Table S3** List of differentially expressed cell cycle- and p53 signalling pathway-related genes with opposite responses to *TNFRSF13B* knockdown between 22Rv1 and PC-3 cells.

| Pathway database | Pathway | Gene name | Gene ID | Fold change in 22Rv1 cells (log2 ratio) | Fold change in PC-3 cells (log2 ratio) |
| --- | --- | --- | --- | --- | --- |
| KEGG | Cell cycle | *BUB1* | 699 | 2.29 | -2.14 |
|  |  | *CCNB1* | 891 | 2.31 | -2.13 |
|  |  | *CDC20* | 991 | 2.91 | -2.58 |
|  |  | *CDC25C* | 995 | 2.01 | -2.57 |
|  |  | *CDC45* | 8318 | 4.25 | -2.56 |
|  |  | *E2F1* | 1869 | 2.39 | -2.06 |
|  |  | *E2F3* | 1871 | 2.24 | -2.24 |
|  |  | *PLK1* | 5347 | 2.14 | -2.41 |
|  | p53 signalling pathway | *CCNB1* | 891 | 2.31 | -2.13 |
|  |  | *PTEN* | 5728 | -2.98 | 2.26 |
|  |  | *RRM2* | 6241 | 2.14 | -2.34 |
| Reactome | Cell cycle | *AURKB* | 9212 | 2.95 | -2.37 |
|  |  | *BUB1* | 699 | 2.29 | -2.14 |
|  |  | *CCNB1* | 891 | 2.31 | -2.13 |
|  |  | *CDC20* | 991 | 2.91 | -2.58 |
|  |  | *CDC25C* | 995 | 2.01 | -2.57 |
|  |  | *CDC45* | 8318 | 4.25 | -2.56 |
|  |  | *CDCA8* | 55143 | 2.08 | -2.76 |
|  |  | *CENPA* | 1058 | 2.72 | -2.04 |
|  |  | *CENPE* | 1062 | 3.50 | -2.28 |
|  |  | *CENPF* | 1063 | 2.24 | -2.73 |
|  |  | *CENPI* | 2491 | 2.99 | -2.42 |
|  |  | *CEP78* | 84131 | 2.37 | -2.19 |
|  |  | *CLSPN* | 63967 | 2.68 | -2.15 |
|  |  | *E2F1* | 1869 | 2.39 | -2.06 |
|  |  | *E2F3* | 1871 | 2.24 | -2.24 |
|  |  | *FBXO5* | 26271 | 2.43 | -2.54 |
|  |  | *H2AC4* | 8335 | 2.68 | -2.69 |
|  |  | *H3C15* | 333932 | 2.06 | -2.88 |
|  |  | *H3C2* | 8358 | 3.87 | -4.59 |
|  |  | *HMMR* | 3161 | 2.53 | -2.06 |
|  |  | *KIF20A* | 10112 | 3.28 | -2.39 |
|  |  | *MIS18A* | 54069 | 2.03 | -2.02 |
|  |  | *MND1* | 84057 | 3.76 | -2.03 |
|  |  | *MYBL2* | 4605 | 2.67 | -2.47 |
|  |  | *NCAPG* | 64151 | 2.71 | -2.88 |
|  |  | *NCAPH* | 23397 | 2.04 | -2.65 |
|  |  | *PLK1* | 5347 | 2.14 | -2.41 |
|  |  | *PLK4* | 10733 | 2.18 | -2.70 |
|  |  | *POLA1* | 5422 | 3.49 | -2.24 |
|  |  | *PRIM1* | 5557 | 2.22 | -2.75 |
|  |  | *PRIM2* | 5558 | 3.23 | -2.23 |
|  |  | *PSMC3IP* | 29893 | 2.69 | -2.32 |
|  |  | *RRM2* | 6241 | 2.14 | -2.34 |
|  |  | *SPC25* | 57405 | 3.77 | -2.14 |
|  |  | *TYMS* | 7298 | 3.83 | -2.02 |
