# Supplementary material for: TNFRSF13B is a potential contributor to prostate cancer
Source: Cancer Cell Int. 2022 May 6;22:180. doi: 10.1186/s12935-022-02590-2 (PMC9074181; doi:10.1186/s12935-022-02590-2)
Supplement: Supplementary file 2 — Additional file 2: The images of the untrimmed western blots. [file 12935_2022_2590_MOESM2_ESM.pptx]

## Slide 1
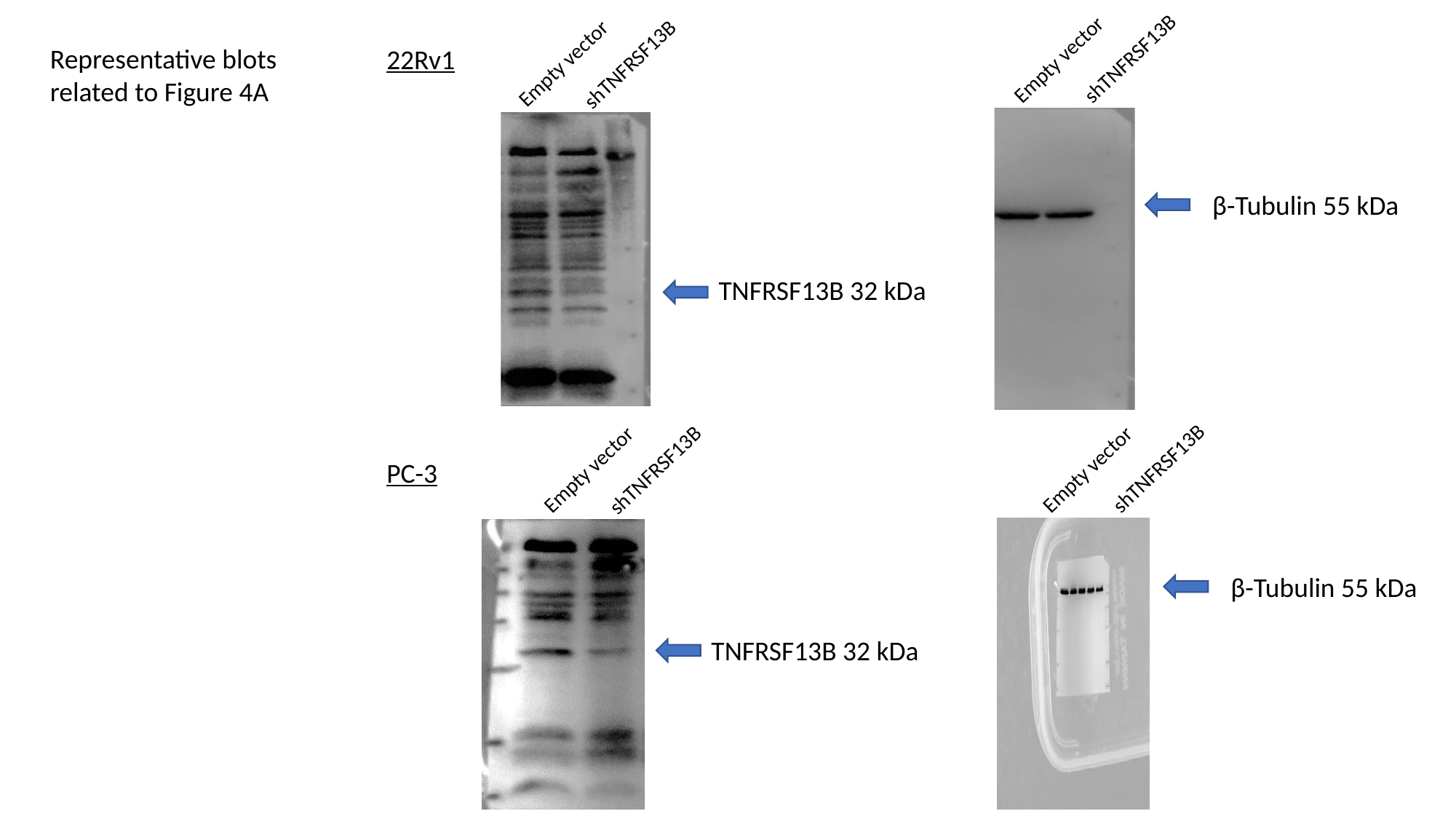

Representative blots
related to Figure 4A
22Rv1
shTNFRSF13B
Empty vector
Empty vector
shTNFRSF13B
β-Tubulin 55 kDa
TNFRSF13B 32 kDa
shTNFRSF13B
PC-3
Empty vector
Empty vector
shTNFRSF13B
β-Tubulin 55 kDa
TNFRSF13B 32 kDa

## Slide 2
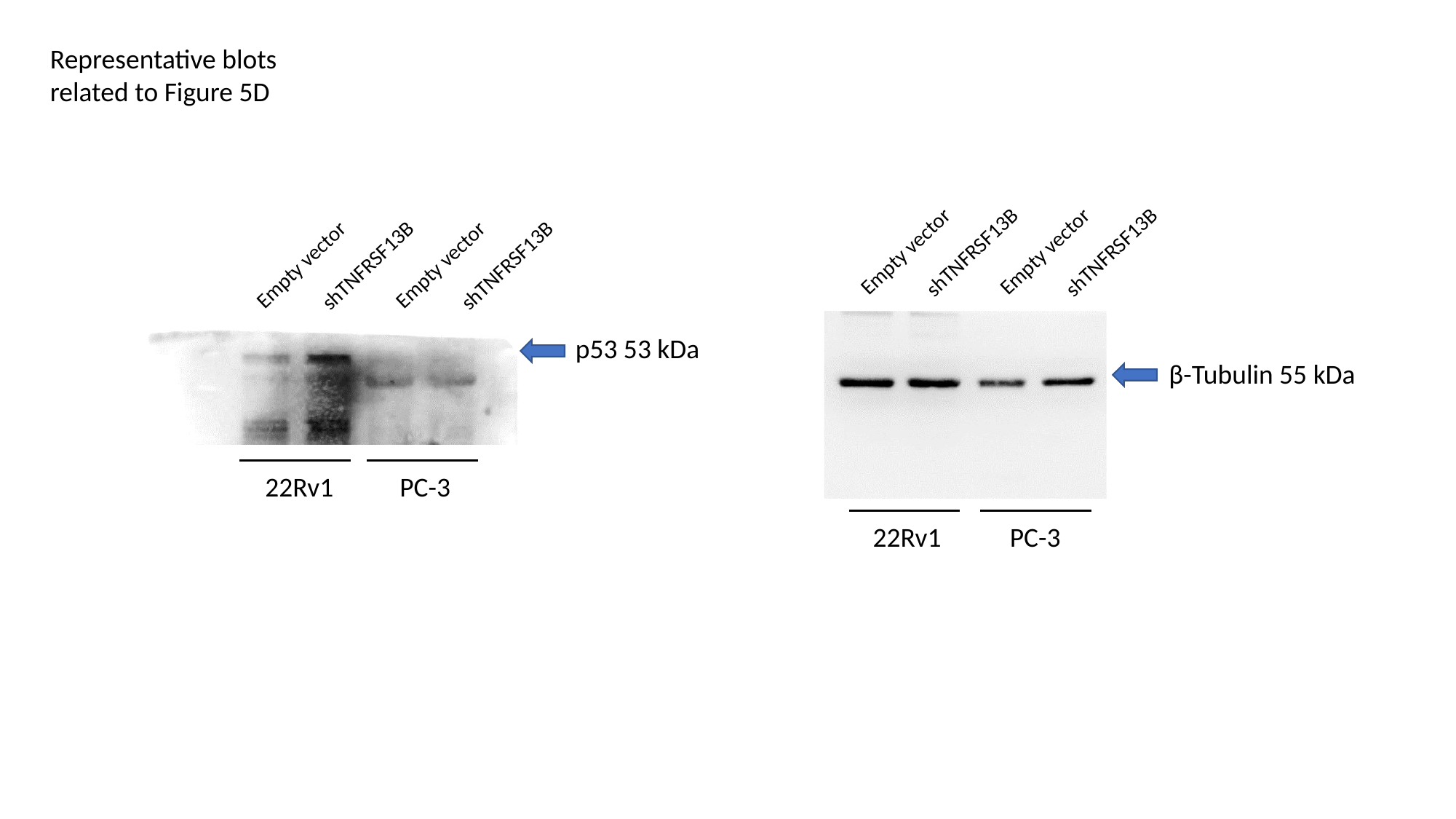

Representative blots
related to Figure 5D
Empty vector
Empty vector
shTNFRSF13B
shTNFRSF13B
Empty vector
Empty vector
shTNFRSF13B
shTNFRSF13B
p53 53 kDa
β-Tubulin 55 kDa
22Rv1
PC-3
22Rv1
PC-3
